# Unknown Circovirus in Immunosuppressed Patient with Hepatitis, France, 2022

**DOI:** 10.3201/eid2905.221485

**Published:** 2023-05

**Authors:** Christophe Rodriguez, Laure Boizeau, Alexandre Soulier, Melissa N’Debi, Vanessa Demontant, Elisabeth Trawinski, Sarah Seng, Hélène Fontaine, Paul-Louis Woerther, Sarah Marchand, Slim Fourati, Stéphane Chevaliez, Pierre Cappy, Stanislas Pol, Jean-Michel Pawlotsky

**Affiliations:** Hôpital Henri Mondor, AP-HP, Université Paris-Est, Créteil, France (C. Rodriguez, L. Boizeau, A. Soulier, M. N’Debi, V. Demontant, E. Trawinski, S. Seng, P.-L. Woerther, S. Marchand, S. Fourati, S. Chevaliez, P. Cappy, J.-M. Pawlotsky);; L’Institut Mondor de Recherche Biomédicale—INSERM U955, Créteil (C. Rodriguez, L. Boizeau, A. Soulier, S. Fourati, S. Chevaliez, P. Cappy, J.-M. Pawlotsky);; Hôpital Cochin, AP-HP, Université Paris-Cité, Paris, France (H. Fontaine, S. Pol)

**Keywords:** Circovirus, hepatitis, shotgun metagenomics, France, viruses

## Abstract

Hepatitis of undetermined origin can be caused by a wide variety of pathogens, sometimes emerging pathogens. We report the discovery, by means of routine shotgun metagenomics, of a new virus belonging to the family Circoviridae, genus *Circovirus*, in a patient in France who had acute hepatitis of unknown origin.

The world is regularly exposed to the emergence or re-emergence of known or unknown infectious agents. The COVID-19 pandemic illustrates the massive impact of such emergence on human lives, national economies, and social organizations. Infections of undetermined origin must be diagnosed early so that adapted measures are put in place to prevent the spread of potentially harmful pathogens. New diagnostic technologies such as shotgun metagenomics (SMg), which requires no prior knowledge of the agents sought, have greatly simplified diagnosis of novel pathogens. SMg has become a key tool for surveillance of viral emergence (*1*). It is regularly used in diagnosing patients with syndromes of suspected viral origin, such as encephalitis, meningitis, pneumopathies, or hepatitis. The Henri Mondor Hospital NGS Plateform laboratory has developed an original SMg technique and has used it for the past 5 years to explore complex infections not diagnosed by classical methods (*2*–*4*). We report detection of a new, yet unknown virus from the family Circoviridae in an immunosuppressed patient with acute hepatitis of unknown origin.

## The Patient

A 61-year-old woman who had undergone heart and lung transplantation for Eisenmenger syndrome 18 years earlier was hospitalized in March 2022 for acute hepatitis of unknown origin. As a result of her immunodepression, she had several infections develop in the preceding 6 months, including ganciclovir-resistant cytomegalovirus (CMV) colitis, parvovirus B19 bicytopenia, and aspergillus bronchitis. At admission, she was receiving multiple therapies, including immunosuppressive and anti-infectious drugs. Serum aminotransferase levels had progressively increased from December 2021 and peaked in April 2022 (alanine aminotransferase, 23× upper limit of normal [ULN]; aspartate aminotransferase, 47× ULN; gamma-glutamyl transpeptidase, 17× ULN; alkaline phosphatase, 1.5× ULN; bilirubin, 54 μmol/L) (Appendix Figure 1).

Results of a liver biopsy showed signs of acute hepatitis, without suggestions of a given etiology. The following markers of infection were absent: hepatitis A virus IgM, hepatitis B virus DNA, hepatitis C virus RNA, hepatitis D virus RNA, hepatitis E virus RNA, HIV RNA, herpes simplex virus 1 and 2 DNA, varicella zoster virus DNA, CMV DNA, Epstein-Barr virus DNA, human herpes virus 6 DNA, adenovirus DNA, enterovirus RNA, parvovirus B19 DNA, and markers of leptospirosis. CMV and Epstein-Barr virus DNAs were undetectable at admission but became detectable at the time of the aminotransferase peak; viral levels were 2.9 log IU/mL for CMV and 4.4 log IU/mL for Epstein-Barr virus. There were no markers of autoimmune hepatitis, and withdrawal or diminution of potentially hepatotoxic treatments had no effect on cytolysis. Aminotransferase levels started to decrease spontaneously 7 weeks after admission. SMg testing was prescribed to identify a potential treatable cause of this acute hepatitis. The patient expressed no opposition to the use of her data and samples for this purpose.

The SMg technique has already been described (*2*–*4*). We performed preextraction mechanical, enzymatic, and chemical actions before extracting both DNAs and RNAs using a DSP DNA Midi Kit on a QiaSymphony device (both QIAGEN, https://www.qiagen.com/us). We generated DNA libraries using a Nextera XT kit nd generated RNA libraries using a TruSeq Total RNA kit (both Illumina, https://www.illumina.com). We sequenced these libraries using NextSeq 500/550 High Output Kit v2.5 300 Cycles (Illumina). We performed metagenomics data analysis using MetaMIC software (https://gitlab.com/mndebi/metamic). The software filters out poor-quality data, identifies sequences by comparison with an nucleotide-based database, reduces background noise by comparison with environmental controls, and establishes a report on the presence or absence of bacteria, viruses, fungi, and parasites.

We performed data reanalysis for genome reconstruction and phylogenetic analysis. We assembled viral DNA sequences and RNA transcripts by using Metaspades 3.15.3 software (*5*). We assembled contigs by means of iterative in-house scripts, gradually replacing the closest reference viral sequences by the patient’s sequences. We checked the consensus sequence by realigning the reads with bwa-mem 0.7.17-r1188 software (https://github.com/lh3/bwa) and by manual checking using the IGV 2.9.4 tool (https://software.broadinstitute.org/software/igv/). We performed phylogenetic analysis using a library of the replicase region and full-length Circovividae genome sequences (*6*), supplemented by the sequences closest to the newly identified virus found using BLASTn (https://blast.ncbi.nlm.nih.gov/Blast.cgi?PROGRAM=tblastn&PAGE_TYPE=BlastSearch&LINK_LOC=blasthome) and the nucleotide database from GenBank, and MUSCLE alignment (*7*) and a maximum-likelihood Kimura model phylogeny by using MEGA5 software (https://www.megasoftware.net).

SMg generated 31,431,784 DNA sequences and 78,933,526 RNA sequences. There were 579,324 DNA sequences and 191,574 RNA sequences related to the DNA genome and RNA transcripts of a yet unknown member of the Circoviridae family, distantly related to *Porcine circovirus 3*. We have provisionally called the new species *Circovirus parisii*.

The viral genome sequence of 2,021 nt could be reconstructed (GenBank accession no. ON526744) (Figure, panel A). The origin of replication located in the AGTATTAC sequence had 1 nucleotide deletion compared with other circoviruses (Figure, panel A). We identified the 2 major circovirus open reading frames (ORFs), starting at positions 140 (replicase, ORF1/rep, sense) and 2,013 (capsid protein, ORF2/cap, antisense), as well as sense ORF3, starting at position 82 (Figure, panel A). The 6 regions described as conserved within the rep region were present, identical to other species from the same genus (Figure, panel A).

**Figure:**
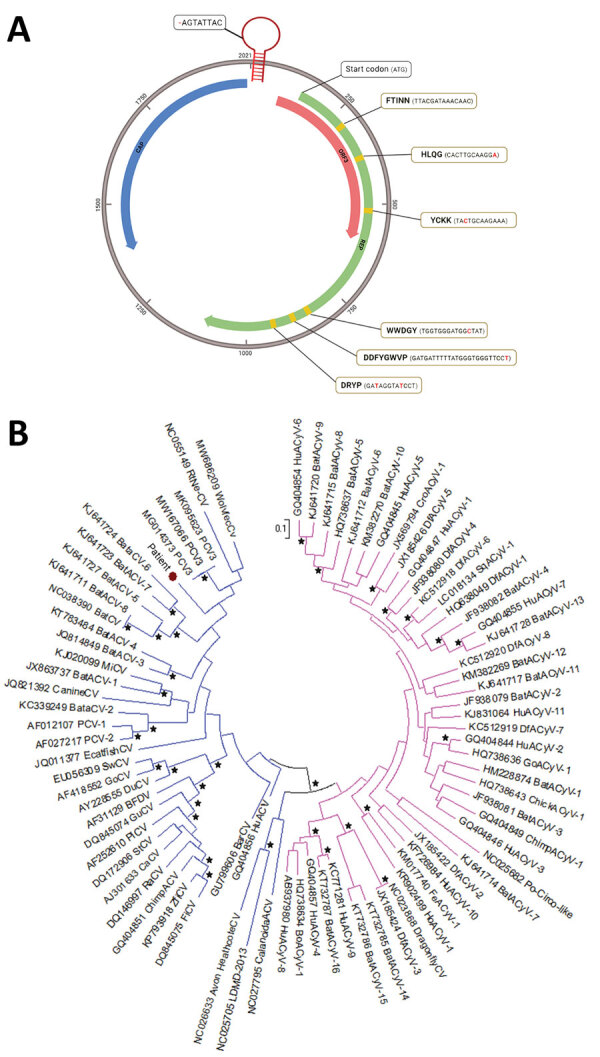
Genomic and phylogenetic analysis of putative novel virus, *Circovirus parisii*, from an immunocompromised patient with hepatitis, France, 2022. A) Full-length genome of *C. parisii* reconstructed from shotgun metagenomics (SMg) sequence analysis. The genome is a 2021-nt single-stranded circular DNA containing 3 predicted open reading frames (ORFs), including ORF1 (replicase, green), ORF2 (capsid protein, blue) and ORF3 (red). The stem-loop contains an AGTATTAC sequence (origin of replication) that misses 1 nt (red dash) compared with other circoviruses. An ATG start codon is located at the 5′ end of the replicase gene. The replicase gene contains 6 conserved motifs, represented with a yellow background (amino acid and nucleotide sequences), with silent substitutions in red. B) Phylogenetic analysis of the replicase gene of the Circoviridae family, including the newly discovered *C. parisii* (red dot), known circoviruses (blue), and known cycloviruses (pink). Bootstrap values >70% are indicated with black stars. Scale bar indicates substitutions per site.

By phylogenetic analysis, the new *C. parisii* clustered with other circoviruses, on the same branch as recently described wolverine circovirus (*8*), rodent circovirus (*9*), and *Porcine circovirus 3*. It was related to another branch containing bat circovirus (Figure, panel B). The genetic distances between *C. parisii* and other circoviruses were of the same order as those between different circovirus species.

The presence of the virus was confirmed by means of a specific PCR technique developed in our lab, which is based on SMg sequencing (Appendix). Sanger sequencing of PCR products yielded a sequence identical to that generated by SMg. No circovirus sequence was found in the environmental control.

## Conclusions

Our shotgun metagenomics approach enabled us to identify a putative new member of the Circoviridae family, provisionally named *C. parisii*, in a profoundly immunosuppressed patient who had self-resolving acute hepatitis. Phylogenetic analysis showed clustering of the new virus with members of the *Circovirus* genus known to infect different animal species. As for other circoviruses, the viral genome displayed an origin of replication (lacking 1 nucleotide), a replicase gene spanning 6 conserved regions, a capsid protein gene, and an ORF3, the role of which remains unknown.

Circoviruses are single-stranded DNA viruses generally transmitted via the fecal–oral route, with a potential pathogenic role in animals. Thus far, no human circovirus infections have been recorded (*10*), and serologic studies have not revealed any human contact (*11*). Nevertheless, culture of *Porcine circovirus 2* on human cell lines, including liver cells, demonstrates the ability of this virus to replicate in human cells (*12*). Various pathologies have been observed in animals infected with circoviruses, including hepatitis (*13*,*14*). *Porcine circovirus 3*, the closest known circovirus, causes respiratory and neurologic diseases, cardiac and multisystemic inflammation, reproductive failure, and porcine dermatitis and nephropathy syndrome (*15*). The presence of the novel virus at the time of the aminotransferase peak raises questions about the causal relationship. Other techniques, such as in situ hybridization on infected tissue, might have offered some insights but were not available in our case. The source of transmission—perhaps animal, perhaps human—could not be established based on this patient’s history.

Appendix Additional information for unknown circovirus in immunosuppressed patient with hepatitis, France, 2022
