# Supplementary material for: Unknown Circovirus in Immunosuppressed Patient with Hepatitis, France, 2022
Source: Emerg Infect Dis. 2023 May;29(5):1051–4. doi: 10.3201/eid2905.221485 (PMC10124635; doi:10.3201/eid2905.221485)
Supplement: Appendix — Additional information for unknown circovirus in immunosuppressed patient with hepatitis, France, 2022 [file 22-1485-Techapp-s1.pdf]

Article DOI: https://doi.org/10.3201/eid2905.221485

EID cannot ensure accessibility for supplementary materials supplied by authors. Readers who have difficulty accessing supplementary content should contact the authors for assistance.

Unknown Circovirus in Immunosuppressed Patient with Hepatitis, France, 2022

Appendix

Specific confirmatory PCR method

Using the viral sequences obtained by SMg, a specific PCR method spanning all the region of the genome has been designed to confirm the presence of the new Circovirus. One step reverse transcription-PCR was performed from 5 µL of blood extract using primers 4 pairs of primers (CiP5s1:GGTTTTCAGGTGTGGCACTT - CiP5as1: CTTGGCCGTTCTAAGTGAG; CiP5s2:GCGAGGTGGTGTTTTACGAT - CiP4as2: GTAATCGCCGTCTTTCTTGC; CiP4s1: CTGCAGTGTGGCAAAGAGAA -CiP3as1: GGAAACACCATCATAGACCTG; CiP3s1: TGCAGAGTTCCACCAGGTTC – CiP1as2: TCTTTTTGGCGGGTCCATC) with the program ([50°C, 60mn; 95°C, 15mn] x 1; [95°C, 30s; 56°C, 30s; 72°C, 1min] x 40; 72°C, 10min) on a Veriti thermocycler (ThermoFisher, Waltham, Massachusetts). A negative control was used for each RT-PCR. PCR products were purified with NucleoFast kit (Macherey-Nagel, Düren, Germany) and sequenced using BigDye Terminator v3.1 Cycle Sequencing Kit (ThermoFisher) on ABI3130 (ThermoFisher).

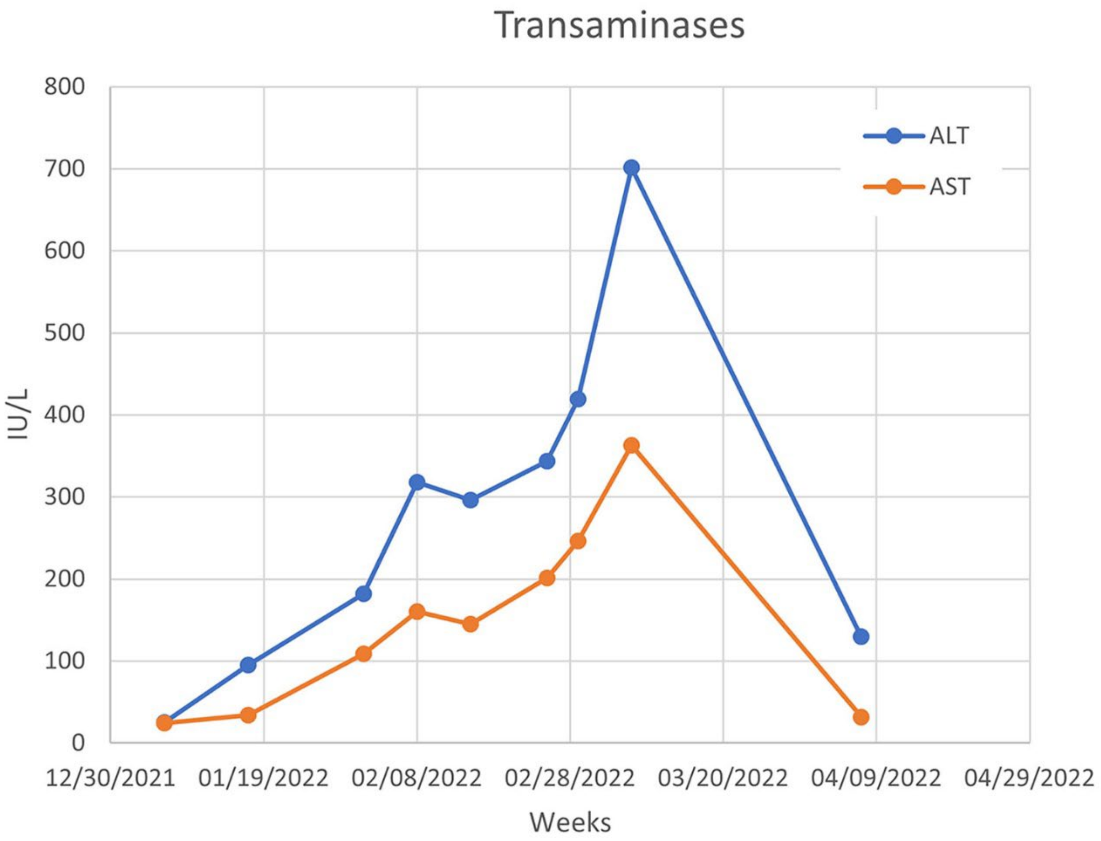

Appendix Figure 1. Kinetics of aminotransferases levels in the patient with acute hepatitis of unknown origin (ALT: alanine aminotransferase, AST: aspartate aminotransferase).

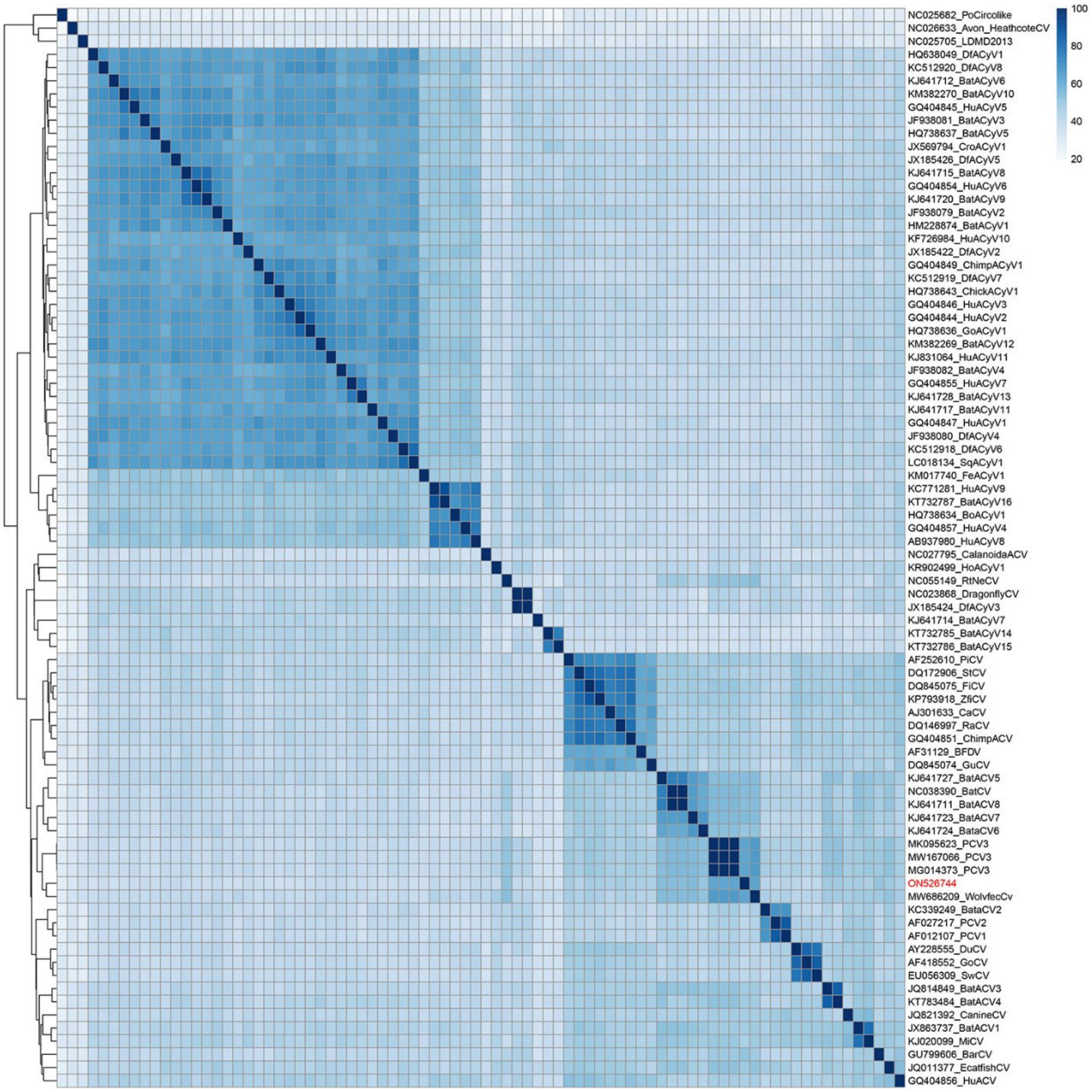

Appendix Figure 2. Genetic identity among known Circovirus strains. The new *Circovirus parisii* is shown in red with its accession number. The blue scale indicates percent genetic identity between strains.
